# Ancient Horizontal Gene Transfer from Bacteria Enhances Biosynthetic Capabilities of Fungi

**DOI:** 10.1371/journal.pone.0004437

**Published:** 2009-02-12

**Authors:** Imke Schmitt, H. Thorsten Lumbsch

**Affiliations:** 1 Department of Plant Biology and Bell Museum of Natural History, University of Minnesota, St. Paul, Minnesota, United States of America; 2 Botany Department, The Field Museum, Chicago, Illinois, United States of America; University of Wisconsin - Madison, United States of America

## Abstract

**Background:**

Polyketides are natural products with a wide range of biological functions and pharmaceutical applications. Discovery and utilization of polyketides can be facilitated by understanding the evolutionary processes that gave rise to the biosynthetic machinery and the natural product potential of extant organisms. Gene duplication and subfunctionalization, as well as horizontal gene transfer are proposed mechanisms in the evolution of biosynthetic gene clusters. To explain the amount of homology in some polyketide synthases in unrelated organisms such as bacteria and fungi, interkingdom horizontal gene transfer has been evoked as the most likely evolutionary scenario. However, the origin of the genes and the direction of the transfer remained elusive.

**Methodology/Principal Findings:**

We used comparative phylogenetics to infer the ancestor of a group of polyketide synthase genes involved in antibiotic and mycotoxin production. We aligned keto synthase domain sequences of all available fungal 6-methylsalicylic acid (6-MSA)-type PKSs and their closest bacterial relatives. To assess the role of symbiotic fungi in the evolution of this gene we generated 24 6-MSA synthase sequence tags from lichen-forming fungi. Our results support an ancient horizontal gene transfer event from an actinobacterial source into ascomycete fungi, followed by gene duplication.

**Conclusions/Significance:**

Given that actinobacteria are unrivaled producers of biologically active compounds, such as antibiotics, it appears particularly promising to study biosynthetic genes of actinobacterial origin in fungi. The large number of 6-MSA-type PKS sequences found in lichen-forming fungi leads us hypothesize that the evolution of typical lichen compounds, such as orsellinic acid derivatives, was facilitated by the gain of this bacterial polyketide synthase.

## Introduction

Polyketides comprise a large class of natural products synthesized by unrelated organisms, such as bacteria, protists, plants, fungi and animals. These compounds are often found in organisms living in mutualistic associations, such as symbiotic bacteria of fungi , insects, and sponges [1]–[4], or lichen-forming fungi [5]. Indeed, lichenized fungi, which maintain obligate associations with cyanobacterial or algal photosynthetic partners are characterized by a sophisticated vegetative morphology and a rich polyketide metabolism [6], [7]. Strikingly, only about 10% of the compounds in the lichen symbiosis occur in other fungi or in vascular plants [8]. The unique secondary metabolism of lichenized fungi exemplifies some of the prevailing problems in natural product research: How did the great diversity of compounds evolve? Which processes initiated the explosive radiation of secondary metabolites in some lineages? To address these issues we employed comparative phylogenetic methods on a set of genes involved in biosynthesis of polyketide extrolites in bacteria, as well as in lichenized and non-lichenized fungi.

Polyketide synthases (PKSs), among other enzymes, are involved in the biosynthesis of polyketides. PKSs are multifunctional enzymes, which are related to fatty acid synthases (FAS) [9], [10]. PKS and FAS condense small carbon units to form the carbon backbone of the polyketide. Structural variation is created by the usage of different starter units and chain extension substrates [11], variable reduction reactions on some or all of the keto groups [10], and post PKS tailoring of the PKS product [12]. Bacteria and fungi commonly harbor a group of PKSs that consists of a single protein complex carrying all catalytic sites (type I PKS). These PKSs are often involved in aromatic polyketide biosynthesis [13]. The domains of type I PKSs may be used reiteratively. A minimal module carries ketosynthase (KS), acyltransferase (AT), and acyl carrier protein (ACP) domains to perform one chain elongation cycle. Optional additional domains responsible for successive reduction steps are ketoreductase (KR), dehydratase (DH), and enoyl reductase (ER). The most conserved gene regions in type I PKS are the KS and AT domains, which are frequently used to infer the evolution of PKS genes [14]–[21]. The phylogenetic placement of the KS can be predictive of some of the PKS's properties, such as reducing or non-reducing functions [16], [22], [23].

The horizontal movement of genetic material between distantly related organisms, horizontal gene transfer (HGT), has played an important role in the evolution of prokaryotes [24] as well as eukaryotes [25], [26]. Interkingdom transfer of genes has also been demonstrated in fungi [27], [28]. While the majority of PKS genes in fungal genomes most likely originated from gene duplication and subsequent subfunctionalization of individual genes [16], a growing body of evidence suggests that HGT has also influenced the evolution of this gene family. Co-regulation of expression has been suggested to be among the causal factors for the clustering of biosynthetic genes in fungi [29], [30]. This clustering facilitates transfer and has been among the arguments for HGT of biosynthetic genes [31]. Further arguments include the location of biosynthetic genes in genome regions that are particularly likely to recombine, such as the telomere ends of the chromosomes [30], and the close proximity to mobile genetic elements [32], [33]. The penicillin cluster, which occurs in many bacteria and a few fungi has been cited as an example of biosynthetic gene HGT. Tentative evidence for this event was found in the codon usage in the fungal penicillin cluster, which is more like that of prokaryotes [34], and in molecular clock estimates on a penicillin cluster phylogeny, which suggest that with respect to the divergence time between bacteria and fungi, the cluster appears much closer than expected to bacterial genes [35].

In the current study we infer the evolutionary history of a clade of fungal type I PKS genes which is closely related to bacterial PKSs. Gene products of PKSs in this clade are small monocyclic or polycyclic aromatic compounds, which are precursors of fungal antibiotics, such as patulin [36], and widespread food-contaminating mycotoxins, such as ochratoxin [37]. Since 6-methylsalicylic acid synthase (6-MSAS) was the first PKS in this group to be characterized [36], we termed this clade “6-MSAS-type PKS”. Gene products of closely related bacterial PKSs include aromatic moieties of potent antibiotics, such as avilamycin [38] and calicheamicin [39]. Since iteratively acting PKSs are rare in bacteria, this group is sometimes referred to as “fungal” type I PKS in bacteria [40]. HGT is typically invoked as the most likely explanation for the phylogenetic placement of the 6-MSAS clade [15], [16], [21]. However, previous studies based this conclusion on tree topology only, and the direction of the interkingdom transfer remained elusive. Kroken et al. (2003) found a clade of fungal PKS genes nested within bacterial sequences and postulated a HGT from bacteria to fungi, while Jenke-Kodama et al. (2005) interpreted the placement of bacterial genes within groups of fungal sequences as evidence for HGT in the opposite direction.

**Figure 1:**
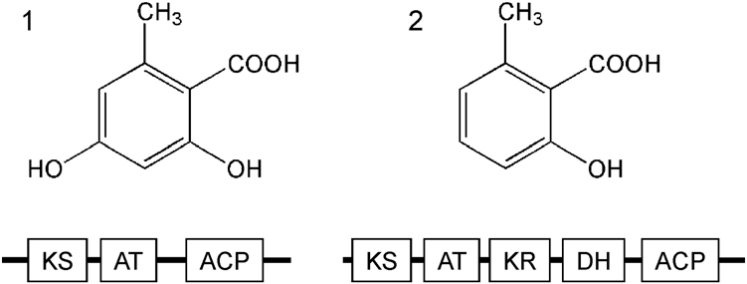
Comparison between orsellinic acid (1) and 6-methylsalicylic acid (6-MSA) (2), and the architecture of the corresponding polyketide synthase genes. Keto synthase (KS), acyl transferase (AT), keto reductase (KR), dehydratase (DH), and acyl carrier protein (ACP) are indicated as boxes. While orsellinic acid has only been characterized in bacteria, 6-MSA synthases with identical architecture have been found in bacteria and ascomycete fungi.

An additional reason for our interest in this clade is the occurrence of sequences from lichenized fungi [41]. While none of the PKS genes found in lichens have yet been functionally characterized, it is possible that 6-MSAS-type genes are involved in lichen compound formation. The lichen-characteristic depsides and depsidones, which result from the coupling of two or more monocyclic polyketides (e.g. orsellinic acid), could be synthesized by a 6-MSAS-type PKS (Daniele Armaleo, personal communication). This can be deduced from the structural similarity of the molecules and the architecture of the genes: 6-MSAS differs from orsellinic acid only in one reduction (Fig. 1). The keto reductase (KR) and dehydratase (DH) domains responsible for this modification could be missing or dysfunctional in the mycobiont PKS, which would then result in the synthesis of orsellinic acid. Furthermore, previous phylogenetic studies have shown that a bacterial orsellinic acid PKS (aviM, AAK83194) is closely related to fungal 6-MSAS-type PKSs [16].

The aim of this study was to establish the phylogenetic origin of the enigmatic fungal 6-MSAS-type PKS biosynthetic gene in a comparative phylogenetic framework. Our results provide statistical support to the hypothesis that this PKS was transferred from an actinobacterial source into ascomycete fungi during an ancient HGT event. We report the finding of 6-MSAS-type PKS genes in a variety of lichen-forming fungi, and speculate about the possible role of lichen symbionts in the evolution of this gene.

## Results

We generated 24 new 6-MSAS-type PKS sequence fragments from lichen-forming fungi (Table 1). In five specimens we found two copies of the gene. Since none of the fungal genomes available to date has two copies of this PKS, and the gene is found only in two of the available ascomycete genomes, we initially questioned the identity of our source material. It is possible that genomic DNA extracts from lichen field collections contain traces of DNA from lichen-associated fungi other than the mycobiont [42], or from lichen-associated bacteria [43]. Theoretically, the 6-MSAS-type PKS copies could stem from genomes other than the mycobiont. However, we can exclude the possibility of a bacterial source, because we found a spliceosomal intron (110 bp, with GT-intron-AG splice sites) in one of the sequences from a lichenized fungus (EF192113). Furthermore, we utilized only those sequences that had the highest BLAST similarity to fungal 6-MSAS-type genes, and discarded all sequences which had the highest similarities to bacterial PKSs. Finally, we are confident that one mycobiont genome can contain multiple copies of the 6-MSAS-type PKS because we recovered two copies each from two axenic mycobiont cultures (*Ochrolechia yasudae* and *Pertusaria corallina*). Sequence divergence between the two copies is 54% in *Ochrolechia yasudae* and 41% in *Pertusaria corallina*. We also retrieved two copies from three herbarium specimens.

**Table 1:** Lichenized fungi used in this study. New sequences are indicated in bold.

Organism	Source	# of PKS found using LC3/LC5c primers	GB accession and clone number
*Baeomyces rufus*	Germany, 24 Jan. 2002, *Zimmermann* (F)	1	**FJ603669 (0758A)**
*Coccotrema cucurbitula*	Argentina, *12 Dec. 2003*, *Messuti&Wirtz* (F)	1	**EF423777 (1438D)**
*Loxosporopsis corallifera*	Canada, *15 June 2004*, *Schmitt* (F)	1	**EF423778 (1476A)**
*Ochrolechia androgyna*	Germany, *15 Apr. 2004*, *Schmitt* (F)	1	**EF423779 (1368A)**
*Ochrolechia yasudae*	Culture 0217M (AKITA)	2	**EF423780 (1724A)**
			**EF423781 (1724E)**
*Pertusaria amara*	Canada, 20 Aug. 2003, *Lumbsch*, *Schmitt*, *Wirtz* (F)	1	**FJ603670 (1066B)**
*Pertusaria aspergilla*	Sweden, *Aug. 2001 Schmitt* (F)	1	**EF423782 (0585A)**
*Pertusaria corallophora*	Antarctica, *Lumbsch 19013d* (F)	1	**EF423783 (1443A)**
*Pertusaria corallophora*	Antarctica, *Lumbsch 19026e* (F)	2	**EF423784 (1446A)**
			**EF423785 (1446C)**
*Pertusaria corallina*	Culture 1118M (AKITA)	2	**EF423786 (1720A)**
			EF192112 (1720f)
*Pertusaria dactylina*	Sweden, *Kanz&Printzen 5435* (HB C.Printzen)	1	**EF423787 (0439B)**
*Pertusaria erythrella*	Australia, *Archer* (ESS 20866)	1	**EF423788 (0326C)**
*Pertusaria excludens*	Spain, *4 June 2003*, *Schmitt* (F)	1	**EF423789 (1022D)**
*Pertusaria hemisphaerica*	Germany, *15 Apr. 2004*, *Schmitt* (F)	1	**EF423790 (1367A)**
*Pertusaria leioplaca*	Czech Republic, *Apr. 2000*, *Schmitt* (F)	2	**EF423791 (0367A)**
			**EF423792 (0367E)**
*Pertusaria mourogana*	Culture 1121M (AKITA)	1	**EF423793 (1721F)**
*Pertusaria ophthalmiza*	Scotland, *Coppins* (ESS 21498)	1	**EF423794 (0631A)**
*Pertusaria pustulata*	Japan, *Yamamoto 15030102* (AKITA)	1	EF192113 (1625f)
*Pertusaria pustulata*	Japan, *Yamamoto 14122626* (AKITA)	2	**EF423795 (1631A)**
			**EF423796 (1631F)**
*Pertusaria scaberula*	Australia, *Archer P932* (NSW)	1	**EF423797 (1448A)**
*Pertusaria subventosa*	Australia, *Lumbsch 19070a* (F)	1	**EF423798 (1078B)**
*Pertusaria subfallens*	Culture 1086M (AKITA)	1	EF192114 (1722f)
*Pertusaria subventosa*	Peru, *Lumbsch*, *Ramirez*, *Wirtz 19351f* (F)	1	EF192115 (1732f)

**Figure 2:**
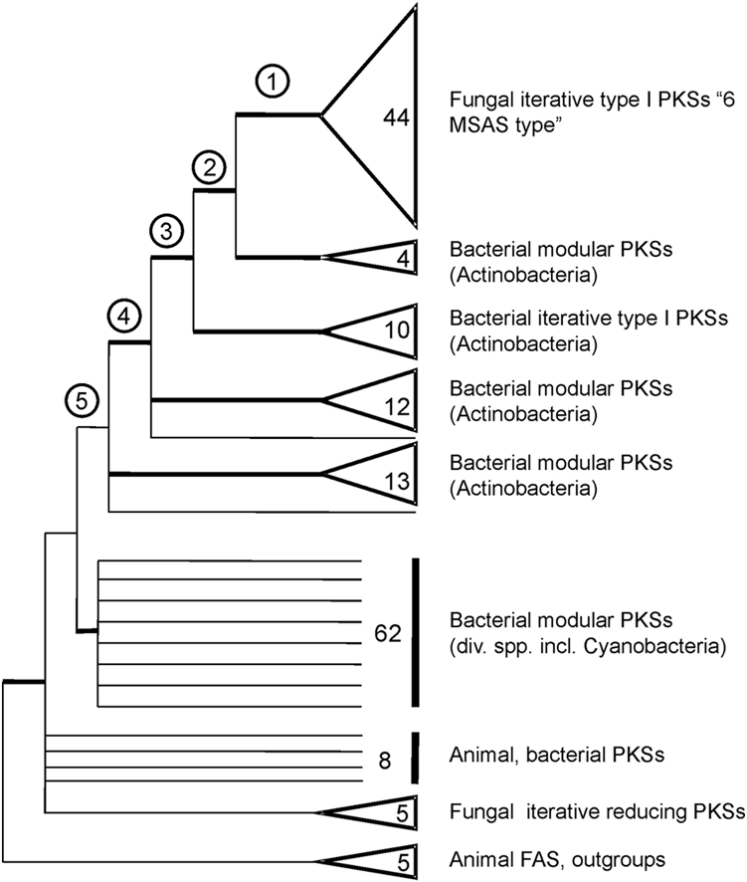
Phylogeny of fungal and bacterial type I PKS genes based on an amino acid alignment of the KS domain (165 sequences). This is a cartoon summary of a 50% majority rule consensus tree of 120,000 trees from a Bayesian analysis. Major clades are collapsed and shown as triangles. Numbers at tips indicate numbers of sequences in the group. Bold branches have significant support (posterior probabilities >94). We reconstructed ancestral character state at nodes 1–5.

Our initial phylogenetic analysis of 165 KS sequences confirms that the fungal 6-MSAS-type clade is more closely related to bacterial than to other fungal PKSs (Fig. 2). This result is in agreement with tree topologies in Kroken et al. (2003) and Castoe et al. (2007). Sister group to the fungal 6-MSAS-type clade is a small group of bacterial modular PKSs (4 sequences), followed by a clade of bacterial iterative type I PKSs (10 sequences). Also closely related are two further clades of bacterial modular PKSs (25 sequences total), which code for enzymes involved in the production of cell-wall-associated lipids in the genus *Mycobacterium*, such as phenolphthiocerol.

**Figure 3:**
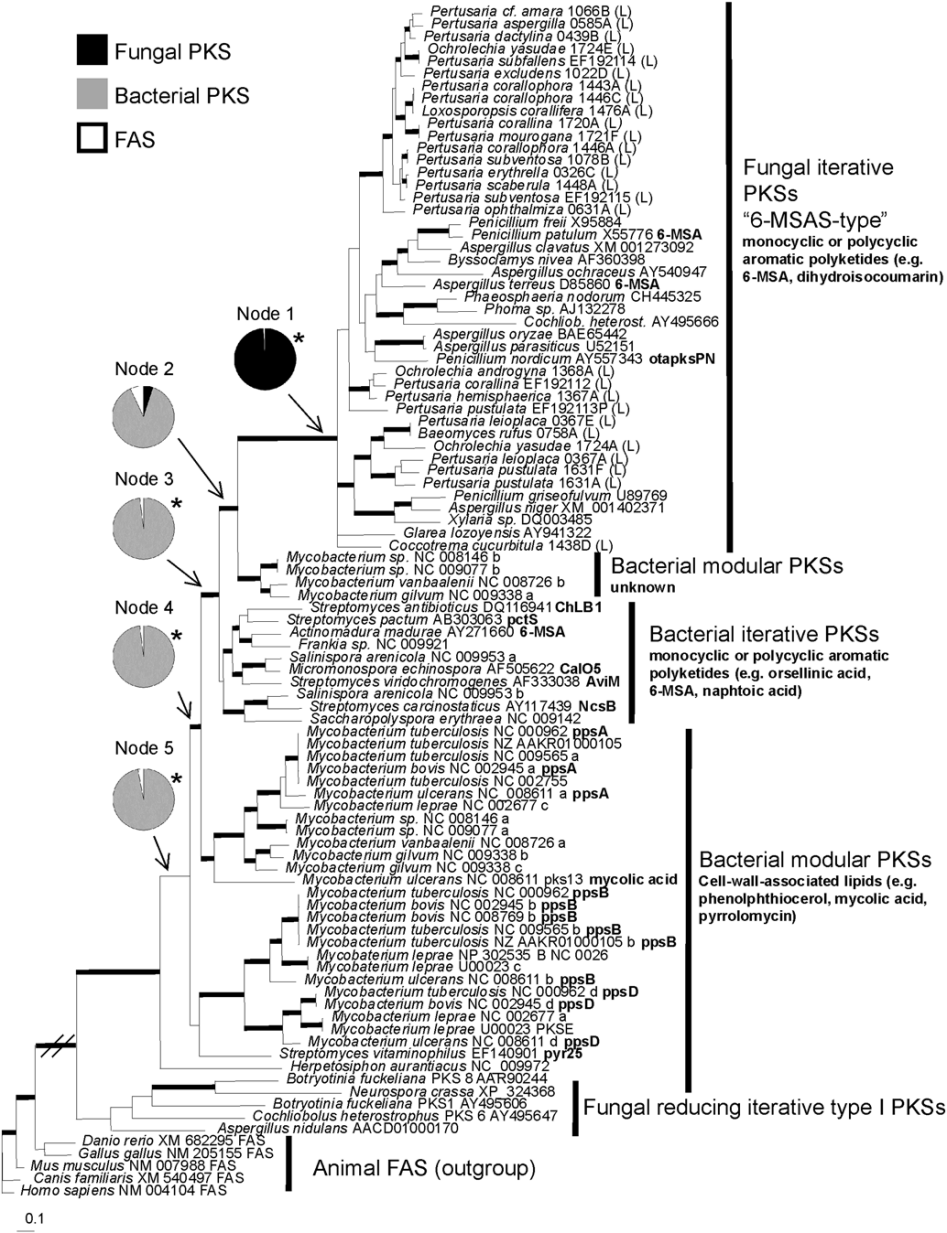
Phylogeny of fungal 6-MSAS-type PKS genes and their bacterial relatives. All PKS belong to the class type I. This is a 50% rule consensus tree from a Bayesian analysis based on a nucleotide alignment of the KS region. Bold branches indicate significant node support (posterior probabilities >94). Pie charts depict likelihood of the ancestor at this node being a fungal PKS (black), bacterial PKS (gray), or a fatty acid synthase (FAS) (white). Asterisks (*) indicate significant probabilities, as shown in Table 2. (L) denotes a lichen-forming ascomycete. Gene designations of characterized genes are indicated in bold print, GenBank accession numbers are given.

To reconstruct the ancestor of the fungal 6-MSAS-type PKS clade, we focused on the four groups of bacterial PKSs that are most closely related to the fungal 6-MSAS-type PKS clade. We analyzed this subset of data based on nucleotide alignments of the KS region. The tree topologies resulting from these alignments are identical to the topology derived from amino acid data. Further, the topologies from three alignments including different outgroups are congruent, and thus only one is shown here (Fig. 3). Results from ancestral character state analyses of three data sets including different outgroups are congruent (Table 2), indicating that outgroup selection has no influence on the reconstructions. The clade most closely related to the fungal 6-MSAS-type PKS consists of four bacterial modular PKSs of unknown function from the genus *Mycobacterium*. Most closely related to these two groups is an unsupported clade of iterative bacterial PKSs. The products of these genes are small aromatic polyketides, such as 6-MSA and orsellinic acid (Fig. 1). We reconstructed the ancestral character states at five selected nodes in this tree (Fig. 3). Our results support a fungal ancestor of the fungal 6-MSAS-type PKS clade (Node 1). For Node 2 we obtained an insignificant result and are unable to infer the ancestral character state. At the deeper nodes (Nodes 3, 4 and 5) we significantly reconstructed bacterial ancestors. These results are consistent with the hypothesis of horizontal gene transfer of the fungal 6-MSAS-type clade from bacteria to fungi [16].

**Table 2:** Ancestral character state reconstructions performed in a combined Bayesian/ML framework.

Node	alignment 1: ingroup+fungal reducing clade	alignment 2: ingroup+FAS	alignment 3: ingroup+fungal reducing+FAS
1	0 = 1.00*	0 = 1.00*	0 = 0.99*
	1 = 0.00*	1 = 0.00*	1 = 0.00*
		2 = 0.00*	2 = 0.01*
2	0 = 0.20	0 = 0.00*	0 = 0.05
	1 = 0.80	1 = 0.97*	1 = 0.88
		2 = 0.03*	2 = 0.07
3	0 = 0.00*	0 = 0.00*	0 = 0.00*
	1 = 1.00*	1 = 0.97*	1 = 0.98*
		2 = 0.03*	2 = 0.02*
4	0 = 0.00*	0 = 0.00*	0 = 0.00*
	1 = 1.00*	1 = 0.97*	1 = 0.98*
		2 = 0.03*	2 = 0.02*
5	0 = 0.00*	0 = 0.00*	0 = 0.00*
	1 = 1.00*	1 = 0.96*	1 = 0.97*
		2 = 0.04*	2 = 0.03*

Characters are coded 0 = fungal PKS, 1 = bacterial PKS, 2 = fatty acid synthase (FAS). Numbers are average probabilities as calculated across all 492 trees in the MCMC sample. Asterisks (*) indicate significant probabilities. Three data sets (alignments 1–3) were analyzed to test possible effects of outgroup selection.

## Discussion

Our results are in agreement with previous studies in suggesting HGT as a likely mechanism in the evolution of fungal 6-MSAS-type PKS genes. Using comparative phylogenetic methods we demonstrated that the origin of this gene is likely to be found in the Actinobacteria. Our analyses suggest that fungal 6-MSAS-type PKS genes came from the ancestor of a group including bacterial iterative PKSs and a small group of bacterial modular PKSs. The wide occurrence of fungal 6-MSAS-type PKSs within the crown group of Ascomycota suggests that the gene was acquired through an ancient HGT event. Fungal 6-MSAS-type PKSs are shared by representatives of different classes within the higher Ascomycota (Leotiomyceta) [44]: Dothideomycetes (*Cochliobolus*, *Phaeosphaeria*), Eurotiomycetes (*Aspergillus*, *Byssochlamys*, *Penicillium*), Lecanoromycetes (all of the lichenized fungi, e.g. *Pertusaria*, *Ochrolechia*), and Sordariomycetes (*Xylaria*). This PKS has not been found in the most basal groups in Ascomycota, such as Saccharomycotina and Taphrinomycotina, or the most basal filamentous fungi (Pezizomycetes). Thus we hypothesize that the PKS was gained through an ancient HGT event that happened before a radiation in Leotiomyceta, which gave rise to the extant crown group of Ascomycota. Subsequently, this horizontally inherited gene was lost in the majority of extant Leotiomyceta. Within some lineages the gene experienced at least one duplication event, as demonstrated by the presence of different copies in some lichenized fungi.

The rare occurrence of introns in fungal 6-MSAS-type PKSs provides additional support to the argument of horizontal transfer from a bacterial source. While we only found a single spliceosomal intron in a 6-MSAS-type PKS from a lichenized fungus, these introns were omnipresent in other types of PKSs from lichenized fungi [18], [45]. The presence of few spliceosomal introns in the 6-MSAS-type PKSs from lichen mycobionts is also consistent with the hypothesis of an ancient HGT in the evolution of Ascomycota: originating from a bacterial source, the gene was free of introns initially, and over time integrated mobile elements from other parts of genome.

There are many examples of intimate bacterial-fungal interactions in nature [46], [47], and HGT between the two kingdoms has been reported [27]. As heterotrophic organisms fungi are often involved in symbiotic relationships, and fossil evidence for such ancient relationships exists [48]. Ancient fungal bacterial interactions have very likely also existed. It is possible that the gene transfer from bacteria to fungi has occurred in such a symbiotic ancestor of Leotiomyceta, the extant crown group of the Ascomycota. As fossil evidence suggests this symbiotic ancestor may have been similar to modern lichenized fungi [49], [50]. Further, phylogenetic studies indicate that major non-lichenized lineages in the Ascomycota were derived from lichen-forming ancestors [51], and recent large scale phylogenies of the Fungi push back the origin of lichenization even further in ascomycete evolution [52]. Biological evidence additionally hints at lichenized fungi as possible organisms for interkingdom gene transfer. Lichens are typically very long lived organisms and may frequently contain cyanobacteria as photosynthetic partners. Indeed, cyanobacterial PKS sequences are among the first BLAST hits when using a fungal 6-MSAS sequence as a query, and they are included in our larger data set (Fig. 2, basal clades). However, cyanobacterial PKSs are not as closely related to the fungal 6-MSAS clade as actinobacterial sequences. Alternatively, the gene may have been transferred within a lichenized ancestor from bacteria other than the primary photobionts. Recent studies report that many lichens contain – in addition to the primary mycobionts and photobionts – a stable consortium of other microorganisms. These include parasitic fungi [53], endophytic fungi [42], and bacteria [43]. Actinobacteria can constitute 10% of the lichen-associated bacterial community [54], and it is conceivable that interkingdom gene transfer was facilitated within this consortium.

The fact that 6-MSAS type PKS genes are only found in a small number of ascomycete genomes suggests that the gene has been lost in most ascomycete species during evolution. This is in agreement with the assumption that secondary metabolite gene clusters, once acquired, are only maintained, if natural selection favors their presence [55]. Interestingly, the 6-MSAS type PKS is found in disproportionally high numbers of lichen-forming fungi, and one might suspect that these genes fulfill an important purpose in these organisms. Biological functions of polyketides in general include signaling, communication, and defense [56]. Specific functions in the lichen symbiosis include UV protection [57], maintenance of the symbiotic equilibrium [58], [59], weathering of rocks for better attachment to the substrate [60], and excretion as stress products [61].

The base unit of depsides and depsidones, which are the most common extrolites found in lichens, is orsellinic or β-orsellinic acid. Considering the structural relatedness of orsellinic acid and 6-MSA (Fig. 1), it seems possible that 6-MSAS-type PKSs from mycobionts code for enzymes that synthesize orsellinic acid. Genetic distances between PKSs in the fungal 6-MSAS-type clade are higher than those in the clade of bacterial iterative PKSs (Fig. 3). Gene products from the bacterial group include the orsellinic acid moieties of calicheamicin [39] and avilamycin [38], as well as the 6-MSA moieties of chlorothricin [62], maduropeptin [63], and pactamycin [64], and the naphtoic acid moiety of neocarzinostatin [65].

Here we could support previous studies suggesting HGT of 6-MSAS-type PKS genes between bacteria and fungi using an extended sampling of DNA sequences. This allowed us to identify actinobacteria as the most likely source for this type of PKS genes in ascomycete fungi. Using comparative phylogenetic analyses, we were able to reject HGT from fungi to bacteria.

## Methods

Material used in this study included mycobiont cultures and field collections of lichens (Table 1). We extracted total genomic DNA, and employed a degenerate primer approach with subsequent cloning to obtain fungal PKS sequences. Primers for PCR amplification were LC3 and LC5c, which preferentially bind to the KS domain of fungal 6MSAS-type PKS genes [22]. Molecular procedures to amplify, clone and sequence these genes are described elsewhere [41]. All sequences were subjected to BLAST searches, and only those with significant homology to fungal 6-MSAS-type PKSs were used for further phylogenetic analysis.

### Alignment and Taxon sampling

Using the LC3/5c primers [22] we obtained two KS paralogs from the mycobiont culture of *Ochrolechia yasudae* (EF423780, EF423781). These sequences were used as query for a BLASTx search in GenBank in April 2008. All sequences which had matches greater than 200 score bits were included in the alignment, and redundant sequences from the two searches were discarded. Additionally, we included five representatives of reducing fungal PKS clades [16], as well as 10 sequences from the “mixed PKS group” [21], because these have been shown to be related to the fungal 6-MSAS group in previous phylogenetic analyses [16], [21]. Five fatty acids (FAS) from animals were used as outgroups. Sequences were aligned based on the amino acid sequence using the program Clustal-W [66]. The alignment included 165 sequences and was 217 amino acids long. A complete list of included sequences is given in Table S1 (supplementary material).

### Phylogenetic analysis

The alignment was analyzed in a Bayesian phylogenetic framework using MrBayes 3.1 [67]. The prior for the amino acid model was set to mixed, allowing the MCMC sampler to explore all of the 10 fixed rate models of amino acid evolution available in MrBayes. Upon convergence of the MCMC procedure each model contributes to the results in proportion to its posterior probability [68]. The program was set to use an invariant gamma distribution, run eight parallel chains, and save every 100^th^ tree. The run was terminated at 6.723.300 generations, when the average standard deviation of split frequencies between the two runs was <0.01, and examination of the p-files in the program Tracer 1.4 (http://tree.bio.ed.ac.uk/software/tracer/) showed that the two runs had converged. The initial 7234 trees were discarded as burn-in of the chain, and the remaining 120.000 (2×60.000) trees were summarized in a 50% majority rule consensus tree. A cartoon summary of this tree is given in Fig. 2.

The tree resulting from this analysis was used to determine the PKS clades most closely related to the fungal 6-MSAS group. The fungal 6-MSAS plus the four most closely related bacterial PKS clades are here referred to as “ingroup”. This subset of sequences was aligned based on amino acid sequences and reverted back to nucleotide sequences. To evaluate potential problems with outgroup selection [69] we compared three alignments including different outgroups: 1. Ingroup+FAS clade, 2. Ingroup+fungal reducing clade, 3. Ingroup+fungal reducing+FAS clades. For each of these data sets ModelTest 3.7 [70] selected the GTR+I+G model of nucleotide evolution. The three alignments were analyzed with MrBayes 3.1. The program was set to run two parallel runs of 5,000,000 generations with 8 chains each, and save every 100^th^ tree into a file. The program Tracer 1.4 was used to determine whether the preset burn-in of 100,000 generations ( = 1,000 trees) was appropriate. The phylogeny resulting from the analysis of alignment 3 is given in Fig. 3.

For ancestral character state reconstructions we used 492 post burn-in trees from the B/MCMC analyses. We sampled every 200^th^ tree ( = every 20,000^th^ generation) to avoid autocorrelation [71]. We employed the program BayesMultistate as implemented in the software BayesTraits (www.evolution.rdg.ac.uk.) to reconstruct ancestral character states in a maximum likelihood framework at five selected nodes in the phylogeny.

## Supporting Information

Table S1 PKS sequences from GenBank included in the alignment. All blastx hits greater 200 score bits were included (EF423780, EF423781 were used as query).
(0.26 MB DOC)
