# Supplementary material for: Ancient Horizontal Gene Transfer from Bacteria Enhances Biosynthetic Capabilities of Fungi
Source: PLoS One. 2009 Feb 12;4(2):e4437. doi: 10.1371/journal.pone.0004437 (PMC2636887; doi:10.1371/journal.pone.0004437)
Supplement: Table S1 — PKS sequences from GenBank included in the alignment. All blastx hits greater 200 score bits were included (EF423780, EF423781 were used as query). (0.26 MB DOC) [file pone.0004437.s001.doc]

| **Table S1. PKS sequences from GenBank included in the alignment. All blastx hits greater 200 score bits were included (EF423780, EF423781 were used as query).** | | | | |
| --- | --- | --- | --- | --- |
| **Species** | **Classification** | **PKS designation, additional information** | **Genbank accession number (nucleotide)** | **GenBank accession number (protein)** |
| *Actinomadura madurae* | Bacteria, Actinobacteria | 6-MSAS, *mdpB* | AY271660 | ABY66019 |
| *Anabaena sp.* | Bacteria, Cyanobacteria |  | AY695349 | AAW55393 |
| *Anabaena sp.* | Bacteria, Cyanobacteria |  | AY695350 | AAW55394 |
| *Anabaena sp.* | Bacteria, Cyanobacteria |  | AY768513 | [AAX44142](http://www.ncbi.nlm.nih.gov/entrez/query.fcgi?cmd=Retrieve&db=Protein&list_uids=61374306&dopt=GenPept&RID=U6BJJ99H015&log$=prottop&blast_rank=93) |
| *Anabaena variabilis* | Bacteria, Cyanobacteria |  | AY768494 | [AAX44125](http://www.ncbi.nlm.nih.gov/entrez/query.fcgi?cmd=Retrieve&db=Protein&list_uids=61374270&dopt=GenPept&RID=U6BJJ99H015&log$=prottop&blast_rank=61) |
| *Anabaena variabilis* | Bacteria, Cyanobacteria |  | NC_007413 a | [YP_324603](http://www.ncbi.nlm.nih.gov/entrez/query.fcgi?cmd=Retrieve&db=Protein&list_uids=75910307&dopt=GenPept&RID=U6C1URGB013&log$=prottop&blast_rank=38) |
| *Anabaena variabilis* | Bacteria, Cyanobacteria |  | NC_007413 b | [YP_325237](http://www.ncbi.nlm.nih.gov/entrez/query.fcgi?cmd=Retrieve&db=Protein&list_uids=75910941&dopt=GenPept&RID=U6BJJ99H015&log$=prottop&blast_rank=62) |
| *Aspergillus clavatus* | Ascomycota, Eurotiomycetes | 6-methylsalicylic acid synthase | XM_001273092 | [XP_001273093](http://www.ncbi.nlm.nih.gov/entrez/query.fcgi?cmd=Retrieve&db=Protein&list_uids=121710954&dopt=GenPept&RID=U6C1URGB013&log$=prottop&blast_rank=9) |
| *Aspergillus nidulans* | Ascomycota, Eurotiomycetes | Outgroup, reducing PKS | AACD01000170 | EAA61508 |
| *Aspergillus niger* | Ascomycota, Eurotiomycetes | PKS | XM_001402371 | [XP_001402408](http://www.ncbi.nlm.nih.gov/entrez/query.fcgi?cmd=Retrieve&db=Protein&list_uids=145256049&dopt=GenPept&RID=U6C1URGB013&log$=prottop&blast_rank=18) |
| *Aspergillus ochraceus* | Ascomycota, Eurotiomycetes | 6-MSAS-type | AY540947 | AAS98200 |
| *Aspergillus oryzae* | Ascomycota, Eurotiomycetes |  | AP007172 | BAE65442 |
| *Aspergillus parasiticus* | Ascomycota, Eurotiomycetes | PKS L2 | U52151 | [AAC23536](http://www.ncbi.nlm.nih.gov/entrez/query.fcgi?cmd=Retrieve&db=Protein&list_uids=1762235&dopt=GenPept&RID=U6C1URGB013&log$=prottop&blast_rank=10) |
| *Aspergillus terreus* | Ascomycota, Eurotiomycetes | atX gene for 6-MSAS | D85860 | BAA20102 |
| *Blastopirellula marina* | Bacteria, Planctomycetes |  | NZ_AANZ01000002 | [ZP_01088963](http://www.ncbi.nlm.nih.gov/entrez/query.fcgi?cmd=Retrieve&db=Protein&list_uids=87306817&dopt=GenPept&RID=U6BJJ99H015&log$=prottop&blast_rank=87) |
| *Bordetella parapertussis* | Bacteria, Proteobacteria | PKS | NC_002928 | gi33594723 |
| *Botryotinia fuckeliana* | Ascomycota, Leotiomycetes | Outgroup, PKS8, reducing PKS | AY495613 | AAR90244 |
| *Botryotinia fuckeliana* | Ascomycota, Leotiomycetes | Outgroup, PKS1, reducing PKS | AY495606 | AAR90237 |
| *Burkholderia mallei* | Bacteria, Proteobacteria | PKS | NC_006348 | gi53723370 |
| *Byssochlamys nivea* | Ascomycota, , Eurotiomycetes | 6-MSAS | AF360398 | AAK48943 |
| *Calothrix desertica* | Bacteria, Cyanobacteria |  | AY695351 | AAW55395 |
| *Calothrix desertica* | Bacteria, Cyanobacteria |  | AY695352 | AAW55396 |
| *Canis familiaris* | Eukaryota, Vertebrata, | Outgroup, FAS | XM_540497 | XP_540497 |
| *Clostridium acetobutylicum* | Bacteria, Firmicutes | PKS | NC_003030 | gi|15893298 |
| *Cochliobolus heterostrophus* | Ascomycota, Dothideomycetes | PKS 25 | AY495666 | AAR90279 |
| *Cochliobolus heterostrophus* | Ascomycota, Dothideomycetes | Outgroup, PKS6, reducing PKS | AY495647 | AAR90261 |
| *Crocosphaera sp.* | Bacteria, Cyanobacteria |  | AY695327 | AAW55371 |
| *Crocosphaera sp.* | Bacteria, Cyanobacteria |  | AY695328 | AAW55372 |
| *Crocosphaera sp.* | Bacteria, Cyanobacteria |  | AY695329 | AAW55373 |
| *Crocosphaera watsonii* | Bacteria, Cyanobacteria |  | NZ_AADV02000002 | [ZP_00514740](http://www.ncbi.nlm.nih.gov/entrez/query.fcgi?cmd=Retrieve&db=Protein&list_uids=67921221&dopt=GenPept&RID=U6BJJ99H015&log$=prottop&blast_rank=104) |
| *Cyanothece sp.* | Bacteria, Cyanobacteria |  | NZ_AAXW01000011 | [ZP_01728763](http://www.ncbi.nlm.nih.gov/entrez/query.fcgi?cmd=Retrieve&db=Protein&list_uids=126657608&dopt=GenPept&RID=U6BJJ99H015&log$=prottop&blast_rank=100) |
| *Cyanothece sp.* | Bacteria, Cyanobacteria |  | NZ_AAXW01000049 | [ZP_01731311](http://www.ncbi.nlm.nih.gov/entrez/query.fcgi?cmd=Retrieve&db=Protein&list_uids=126660194&dopt=GenPept&RID=U6BJJ99H015&log$=prottop&blast_rank=52) |
| *Danio rerio* | Eukaryota, Vertebrata, Teleostei | PKS | BX005238 | gi35209055 |
| *Danio rerio* | Eukaryota, Vertebrata, | Outgroup, FAS | XM_682295 | XP_687387 |
| *Frankia sp.* | Bacteria, Actinobacteria | Iterative type I | NC_009921 | [YP_001507916](http://www.ncbi.nlm.nih.gov/entrez/query.fcgi?cmd=Retrieve&db=Protein&list_uids=158315408&dopt=GenPept&RID=U6C1URGB013&log$=prottop&blast_rank=27) |
| *Gallus gallus* | Eukaryota, Vertebrata, | Outgroup, FAS | NM_205155 | NP_990486 |
| *Glarea lozoyensis* | Ascomycota, mitosporic Ascomycota |  | AY941322 | AAX35547 |
| *Gloeothece sp.* | Bacteria, Cyanobacteria |  | AY768476 | [AAX44107](http://www.ncbi.nlm.nih.gov/entrez/query.fcgi?cmd=Retrieve&db=Protein&list_uids=61374234&dopt=GenPept&RID=U6BJJ99H015&log$=prottop&blast_rank=63) |
| *Herpetosiphon aurantiacus* | Bacteria, Chloroflexi |  | NC_009972 | YP_001545180 |
| Herpetosiphon aurantiacus | Bacteria, Chloroflexi |  | NC_009972 | [YP_001544628](http://www.ncbi.nlm.nih.gov/entrez/query.fcgi?cmd=Retrieve&db=Protein&list_uids=159898381&dopt=GenPept&RID=U6BJJ99H015&log$=prottop&blast_rank=118) |
| *Homo sapiens* | Eukaryota, Vertebrata | Outgroup, FAS | U26644 | AAC50259 |
| *Leptolynya sp.* | Bacteria, Cyanobacteria |  | AY604658 | AAU93833 |
| *Lynya majuscula* | Bacteria, Cyanobacteria; | JamE , jamaicamide | AY522504 JamE | AAS98777 |
| *Lynya majuscula* | Bacteria, Cyanobacteria; | JamL , jamaicamide | AY522504 JamL | AAS98783 |
| *Lynya majuscula* | Bacteria, Cyanobacteria | CurJ , jamaicamide | AY652953 CurJ | AAT70105 |
| *Lynya majuscula* | Bacteria, Cyanobacteria | CurK , jamaicamide | AY652953 CurK | AAT70106 |
| *Lynya majuscula* | Bacteria, Cyanobacteria | HctD , hectochlorin | AY974560 | [AAY42396](http://www.ncbi.nlm.nih.gov/entrez/query.fcgi?cmd=Retrieve&db=Protein&list_uids=65336267&dopt=GenPept&RID=U6BJJ99H015&log$=prottop&blast_rank=80) |
| *Microbulbifer degradans* | Bacteria, Proteobacteria | PKS | NZ_AABI03000004 | gi48862361 |
| *Microcoleus chthonoplastes* | Bacteria, Cyanobacteria |  | AY695342 | AAW55386 |
| *Microcoleus chthonoplastes* | Bacteria, Cyanobacteria |  | AY695343 | AAW55387 |
| *Microcoleus chthonoplastes* | Bacteria, Cyanobacteria |  | AY695344 | AAW55388 |
| *Microcoleus sp.* | Bacteria, Cyanobacteria |  | AY768500 | [AAX44130](http://www.ncbi.nlm.nih.gov/entrez/query.fcgi?cmd=Retrieve&db=Protein&list_uids=61374281&dopt=GenPept&RID=U6BJJ99H015&log$=prottop&blast_rank=83) |
| *Microcystis* | Bacteria, Cyanobacteria |  | NC_010296 | [YP_001657795](http://www.ncbi.nlm.nih.gov/entrez/query.fcgi?cmd=Retrieve&db=Protein&list_uids=166365522&dopt=GenPept&RID=U6BJJ99H015&log$=prottop&blast_rank=56) |
| *Microcystis aeruginosa* | bacteria | psm3M, NRPS | AB279593 | BAF68998 |
| *Micromonospora echinospora* | Bacteria, Actinobacteria | CalO5, Iterative type I | AF497482 | AAM70355 |
| *Mus musculus* | Eukaryota, Vertebrata, | Outgroup, FAS | NM_007988 | NP_032014 |
| *Mycobacterium bovis* | Bacteria, Actinobacteria | ppsA, phenolphthiocerol | NC_002945 ppsA | [NP_856601](http://www.ncbi.nlm.nih.gov/entrez/query.fcgi?cmd=Retrieve&db=Protein&list_uids=31794108&dopt=GenPept&RID=U6BJJ99H015&log$=prottop&blast_rank=46) |
| *Mycobacterium bovis* | Bacteria, Actinobacteria | ppsB, phenolphthiocerol | NC_002945 ppsB | [NP_856602](http://www.ncbi.nlm.nih.gov/entrez/query.fcgi?cmd=Retrieve&db=Protein&list_uids=31794109&dopt=GenPept&RID=U6BJJ99H015&log$=prottop&blast_rank=73) |
| *Mycobacterium bovis* | Bacteria, Actinobacteria | ppsD, phenolphthiocerol | NC_002945 ppsD | [NP_856604](http://www.ncbi.nlm.nih.gov/entrez/query.fcgi?cmd=Retrieve&db=Protein&list_uids=31794111&dopt=GenPept&RID=U6BJJ99H015&log$=prottop&blast_rank=51) |
| *Mycobacterium bovis* | Bacteria, Actinobacteria | ppsB, phenolpthiocerol | NC_008769 | [YP_979038](http://www.ncbi.nlm.nih.gov/entrez/query.fcgi?cmd=Retrieve&db=Protein&list_uids=121638814&dopt=GenPept&RID=U6BJJ99H015&log$=prottop&blast_rank=71) |
| *Mycobacterium gilvum* | Bacteria, Actinobacteria |  | NC_009338 a | [YP_001133955](http://www.ncbi.nlm.nih.gov/entrez/query.fcgi?cmd=Retrieve&db=Protein&list_uids=145223277&dopt=GenPept&RID=U6C1URGB013&log$=prottop&blast_rank=24) |
| *Mycobacterium gilvum* | Bacteria, Actinobacteria | modular | NC_009338 b | [YP_001134657](http://www.ncbi.nlm.nih.gov/entrez/query.fcgi?cmd=Retrieve&db=Protein&list_uids=145223979&dopt=GenPept&RID=U6BJJ99H015&log$=prottop&blast_rank=90) |
| *Mycobacterium gilvum* | Bacteria, Actinobacteria | modular | NC_009338 c | [YP_001134659](http://www.ncbi.nlm.nih.gov/entrez/query.fcgi?cmd=Retrieve&db=Protein&list_uids=145223981&dopt=GenPept&RID=U6BJJ99H015&log$=prottop&blast_rank=94) |
| *Mycobacterium leprae* | Bacteria, Actinobacteria |  | NC_002677 a | [NP_302533](http://www.ncbi.nlm.nih.gov/entrez/query.fcgi?cmd=Retrieve&db=Protein&list_uids=15828270&dopt=GenPept&RID=U6BJJ99H015&log$=prottop&blast_rank=50) |
| *Mycobacterium leprae* | Bacteria, Actinobacteria |  | NC_002677 b | [NP_302535](http://www.ncbi.nlm.nih.gov/entrez/query.fcgi?cmd=Retrieve&db=Protein&list_uids=15828272&dopt=GenPept&RID=U6BJJ99H015&log$=prottop&blast_rank=69) |
| *Mycobacterium leprae* | Bacteria, Actinobacteria |  | NC_002677 c | [NP_302536](http://www.ncbi.nlm.nih.gov/entrez/query.fcgi?cmd=Retrieve&db=Protein&list_uids=15828273&dopt=GenPept&RID=U6BJJ99H015&log$=prottop&blast_rank=35) |
| *Mycobacterium leprae* | Bacteria, Actinobacteria | pksC | U00023 pksC | [AAA17356](http://www.ncbi.nlm.nih.gov/entrez/query.fcgi?cmd=Retrieve&db=Protein&list_uids=467199&dopt=GenPept&RID=U6BJJ99H015&log$=prottop&blast_rank=67) |
| *Mycobacterium leprae* | Bacteria, Actinobacteria | pksE | U00023 pksE | [AAA17364](http://www.ncbi.nlm.nih.gov/entrez/query.fcgi?cmd=Retrieve&db=Protein&list_uids=467207&dopt=GenPept&RID=U6BJJ99H015&log$=prottop&blast_rank=49) |
| *Mycobacterium sp.* | Bacteria, Actinobacteria | Mmcs_2835 | NC_008146 a | [YP_639998](http://www.ncbi.nlm.nih.gov/entrez/query.fcgi?cmd=Retrieve&db=Protein&list_uids=108799801&dopt=GenPept&RID=U6BJJ99H015&log$=prottop&blast_rank=37) |
| *Mycobacterium sp.* | Bacteria, Actinobacteria |  | NC_008146 b | [YP_640635](http://www.ncbi.nlm.nih.gov/entrez/query.fcgi?cmd=Retrieve&db=Protein&list_uids=108800438&dopt=GenPept&RID=U6C1URGB013&log$=prottop&blast_rank=23) |
| *Mycobacterium sp.* | Bacteria, Actinobacteria |  | NC_009077 a | [YP_001071136](http://www.ncbi.nlm.nih.gov/entrez/query.fcgi?cmd=Retrieve&db=Protein&list_uids=126435445&dopt=GenPept&RID=U6BJJ99H015&log$=prottop&blast_rank=36) |
| *Mycobacterium sp.* | Bacteria, Actinobacteria |  | NC_009077 b | [YP_001071753](http://www.ncbi.nlm.nih.gov/entrez/query.fcgi?cmd=Retrieve&db=Protein&list_uids=126436062&dopt=GenPept&RID=U6C1URGB013&log$=prottop&blast_rank=22) |
| *Mycobacterium tuberculosis* | Bacteria, Actinobacteria |  | NC_002755 | [NP_337514](http://www.ncbi.nlm.nih.gov/entrez/query.fcgi?cmd=Retrieve&db=Protein&list_uids=15842477&dopt=GenPept&RID=U6BJJ99H015&log$=prottop&blast_rank=41) |
| *Mycobacterium tuberculosis* | Bacteria, Actinobacteria | ppsA, phenolpthiocerol | NC_009565 ppsA | [YP_001288874](http://www.ncbi.nlm.nih.gov/entrez/query.fcgi?cmd=Retrieve&db=Protein&list_uids=148824120&dopt=GenPept&RID=U6BJJ99H015&log$=prottop&blast_rank=44) |
| *Mycobacterium tuberculosis* | Bacteria, Actinobacteria | ppsB, phenolpthiocerol | NC_009565 ppsB | [YP_001288875](http://www.ncbi.nlm.nih.gov/entrez/query.fcgi?cmd=Retrieve&db=Protein&list_uids=148824121&dopt=GenPept&RID=U6BJJ99H015&log$=prottop&blast_rank=70) |
| *Mycobacterium tuberculosis* | Bacteria, Actinobacteria |  | NZ_AAKR01000105 a | [ZP_00877561](http://www.ncbi.nlm.nih.gov/entrez/query.fcgi?cmd=Retrieve&db=Protein&list_uids=81252993&dopt=GenPept&RID=U6BJJ99H015&log$=prottop&blast_rank=43) |
| *Mycobacterium tuberculosis* | Bacteria, Actinobacteria |  | NZ_AAKR01000105 b | [ZP_00877563](http://www.ncbi.nlm.nih.gov/entrez/query.fcgi?cmd=Retrieve&db=Protein&list_uids=81252995&dopt=GenPept&RID=U6BJJ99H015&log$=prottop&blast_rank=75) |
| *Mycobacterium tuberculosis H37Rv* | Bacteria, Actinobacteria | ppsA, phenolphthiocerol | NC_000962 ppsA | [NP_217447](http://www.ncbi.nlm.nih.gov/entrez/query.fcgi?cmd=Retrieve&db=Protein&list_uids=15610068&dopt=GenPept&RID=U6BJJ99H015&log$=prottop&blast_rank=42) |
| *Mycobacterium tuberculosis H37Rv* | Bacteria, Actinobacteria | ppsB, phenolphthiocerol | NC_000962 ppsB | [NP_217448](http://www.ncbi.nlm.nih.gov/entrez/query.fcgi?cmd=Retrieve&db=Protein&list_uids=15610069&dopt=GenPept&RID=U6BJJ99H015&log$=prottop&blast_rank=74) |
| *Mycobacterium tuberculosis H37Rv* | Bacteria, Actinobacteria | ppsD, phenolphthiocerol | NC_000962 ppsD | [NP_217450](http://www.ncbi.nlm.nih.gov/entrez/query.fcgi?cmd=Retrieve&db=Protein&list_uids=15610071&dopt=GenPept&RID=U6BJJ99H015&log$=prottop&blast_rank=53) |
| *Mycobacterium ulcerans* | Bacteria, Actinobacteria | PKS13, mycolic acids | NC_008611 pks13 | [YP_908332](http://www.ncbi.nlm.nih.gov/entrez/query.fcgi?cmd=Retrieve&db=Protein&list_uids=118620000&dopt=GenPept&RID=U6BJJ99H015&log$=prottop&blast_rank=111) |
| *Mycobacterium ulcerans* | Bacteria, Actinobacteria | Phenolpthiocerol ppsA  phthiodiolones (*ppsA-E*) | NC_008611 ppsA | [YP_905929](http://www.ncbi.nlm.nih.gov/entrez/query.fcgi?cmd=Retrieve&db=Protein&list_uids=118617597&dopt=GenPept&RID=U6BJJ99H015&log$=prottop&blast_rank=34) |
| *Mycobacterium ulcerans* | Bacteria, Actinobacteria | Phenolpthiocerol ppsB | NC_008611 ppsB | [YP_905928](http://www.ncbi.nlm.nih.gov/entrez/query.fcgi?cmd=Retrieve&db=Protein&list_uids=118617596&dopt=GenPept&RID=U6BJJ99H015&log$=prottop&blast_rank=82) |
| *Mycobacterium ulcerans* | Bacteria, Actinobacteria | ppsD, phenolpthiocerol | NC_008611 ppsD | [YP_905926](http://www.ncbi.nlm.nih.gov/entrez/query.fcgi?cmd=Retrieve&db=Protein&list_uids=118617594&dopt=GenPept&RID=U6BJJ99H015&log$=prottop&blast_rank=101) |
| *Mycobacterium vanbaalenii* | Bacteria, Actinobacteria |  | NC_008726 a | [YP_953934](http://www.ncbi.nlm.nih.gov/entrez/query.fcgi?cmd=Retrieve&db=Protein&list_uids=120404105&dopt=GenPept&RID=U6BJJ99H015&log$=prottop&blast_rank=92) |
| *Mycobacterium vanbaalenii* | Bacteria, Actinobacteria | Clade 2, Mvan_3852  Clade 2 KS-AT-KR-PP | NC_008726 b | [YP_954639](http://www.ncbi.nlm.nih.gov/entrez/query.fcgi?cmd=Retrieve&db=Protein&list_uids=120404810&dopt=GenPept&RID=U6C1URGB013&log$=prottop&blast_rank=25) |
| *Myxococcus xanthus* | Bacteria, Proteobacteria | NRPS | NC_008095 | [YP_632575](http://www.ncbi.nlm.nih.gov/entrez/query.fcgi?cmd=Retrieve&db=Protein&list_uids=108763177&dopt=GenPept&RID=U6BJJ99H015&log$=prottop&blast_rank=86) |
| *Neurospora crassa* | Ascomycota, Sordariomycetes | Outgroup, reducing PKS | XM_324367 | XP_324368 |
| *Nitrosomonas europaea* | Bacteria, Proteobacteria | PKS | NC_004757 | gi30248031 |
| *Nocardia farcinica* | Bacteria, Actinobacteria | PKS | NC_006361 | gi54021964 |
| *Nodularia harveyana* | Bacteria, Cyanobacteria |  | AY768516 | [AAX44145](http://www.ncbi.nlm.nih.gov/entrez/query.fcgi?cmd=Retrieve&db=Protein&list_uids=61374312&dopt=GenPept&RID=U6BJJ99H015&log$=prottop&blast_rank=106) |
| *Nostoc punctiforme* | Bacteria, Cyanobacteria |  | AY695331 | AAW55375 |
| *Nostoc punctiforme* | Bacteria, Cyanobacteria |  | AY695341 | AAW55385 |
| *Nostoc punctiforme* | Bacteria, Cyanobacteria |  | NZ_AAAY02000050 a | [ZP_00108802](http://www.ncbi.nlm.nih.gov/entrez/query.fcgi?cmd=Retrieve&db=Protein&list_uids=23126921&dopt=GenPept&RID=U6BJJ99H015&log$=prottop&blast_rank=84) |
| *Nostoc punctiforme* | Bacteria, Cyanobacteria |  | NZ_AAAY02000050 b | [ZP_00110105](http://www.ncbi.nlm.nih.gov/entrez/query.fcgi?cmd=Retrieve&db=Protein&list_uids=23128252&dopt=GenPept&RID=U6BJJ99H015&log$=prottop&blast_rank=78) |
| *Nostoc punctiforme* | Bacteria, Cyanobacteria |  | NZ_AAAY02000050 c | [ZP_00110106](http://www.ncbi.nlm.nih.gov/entrez/query.fcgi?cmd=Retrieve&db=Protein&list_uids=23128253&dopt=GenPept&RID=U6BJJ99H015&log$=prottop&blast_rank=99) |
| *Nostoc sp.* | Bacteria, Cyanobacteria |  | AY695345 | AAW55389 |
| *Nostoc sp.* | Bacteria, Cyanobacteria |  | NC_003272 | [NP_485688](http://www.ncbi.nlm.nih.gov/entrez/query.fcgi?cmd=Retrieve&db=Protein&list_uids=17229140&dopt=GenPept&RID=U6BJJ99H015&log$=prottop&blast_rank=68) |
| *Penicillium freii* | Ascomycota, Eurotiomycetes |  | X95884 | CAA65133 |
| *Penicillium griseofulvum* | Ascomycota, Eurotiomycetes |  | U89769 | AAB49684 |
| *Penicillium nordicum* | Ascomycota, Eurotiomycetes | otapksPN, ochratoxin A | AY557343 | AAP33839.2 |
| *Penicillium patulum* | Ascomycota, Eurotiomycetes | 6-MSAS | X55776 | P22367 |
| *Pertuaria subventosa* | Ascomycota, Lecanoromycetes |  | EF192115 | [ABQ11384](http://www.ncbi.nlm.nih.gov/entrez/query.fcgi?cmd=Retrieve&db=Protein&list_uids=146220629&dopt=GenPept&RID=U6C1URGB013&log$=prottop&blast_rank=2) |
| *Pertusaria corallina* | Ascomycota, Lecanoromycetes |  | EF192112 | [ABQ11381](http://www.ncbi.nlm.nih.gov/entrez/query.fcgi?cmd=Retrieve&db=Protein&list_uids=146220623&dopt=GenPept&RID=U6C1URGB013&log$=prottop&blast_rank=7) |
| *Pertusaria pustulata* | Ascomycota, Lecanoromycetes |  | EF192113 | [ABQ11382](http://www.ncbi.nlm.nih.gov/entrez/query.fcgi?cmd=Retrieve&db=Protein&list_uids=146220625&dopt=GenPept&RID=U6C1URGB013&log$=prottop&blast_rank=6) |
| *Pertusaria subfallens* | Ascomycota, Lecanoromycetes |  | EF192114 | [ABQ11383](http://www.ncbi.nlm.nih.gov/entrez/query.fcgi?cmd=Retrieve&db=Protein&list_uids=146220627&dopt=GenPept&RID=U6C1URGB013&log$=prottop&blast_rank=1) |
| Phaeosphaeria nodorum | Ascomycota; Dothideomycetes |  | CH445325 | EAT91972 |
| *Phoma sp.* | Ascomycota, Dothideomycetes |  | AJ132278 | CAB44720 |
| *Pleurocapsa sp.* | Bacteria, Cyanobacteria |  | AY695339 | AAW55383 |
| *Pleurocapsa sp.* | bacteria |  | AY695347 | AAW55391 |
| *Pleurocapsa sp.* | bacteria |  | AY695348 | AAW55392 |
| *Polyangium cellulosum* | Bacteria, Proteobacteria |  | DQ359866 | [ABD17628](http://www.ncbi.nlm.nih.gov/entrez/query.fcgi?cmd=Retrieve&db=Protein&list_uids=87045582&dopt=GenPept&RID=U6BJJ99H015&log$=prottop&blast_rank=95) |
| *Polyangium cellulosum* | Bacteria, Proteobacteria |  | DQ359867 | [ABD17629](http://www.ncbi.nlm.nih.gov/entrez/query.fcgi?cmd=Retrieve&db=Protein&list_uids=87045584&dopt=GenPept&RID=U6BJJ99H015&log$=prottop&blast_rank=85) |
| *Rhodopirellula baltica* | Bacteria, Planctomycetes | PKS | NC_005027 | gi32470666 |
| *Saccharopolyspora erythraea* | Bacteria, Actinobacteria | Iterative type I | NC_009142 | [YP_001107644](http://www.ncbi.nlm.nih.gov/entrez/query.fcgi?cmd=Retrieve&db=Protein&list_uids=134101983&dopt=GenPept&RID=U6C1URGB013&log$=prottop&blast_rank=35) |
| *Salinispora arenicola* | Bacteria, Actinobacteria | Iterative type I | NC_009953 a | [YP_001536890](http://www.ncbi.nlm.nih.gov/entrez/query.fcgi?cmd=Retrieve&db=Protein&list_uids=159037637&dopt=GenPept&RID=U6C1URGB013&log$=prottop&blast_rank=30) |
| *Salinispora arenicola* | Bacteria, Actinobacteria | Iterative type I | NC_009953 b | [YP_001539688](http://www.ncbi.nlm.nih.gov/entrez/query.fcgi?cmd=Retrieve&db=Protein&list_uids=159040435&dopt=GenPept&RID=U6C1URGB013&log$=prottop&blast_rank=34) |
| *Scytonema* | bacteria Cyanobacteria, |  | AY695321 | AAW55365 |
| *Scytonema sp.* | Bacteria, Cyanobacteria |  | AY695358 | [AAW55402](http://www.ncbi.nlm.nih.gov/entrez/query.fcgi?cmd=Retrieve&db=Protein&list_uids=57638729&dopt=GenPept&RID=U6BJJ99H015&log$=prottop&blast_rank=116) |
| *Scytonema sp.* | Bacteria, Cyanobacteria |  | AY695359 | [AAW55403](http://www.ncbi.nlm.nih.gov/entrez/query.fcgi?cmd=Retrieve&db=Protein&list_uids=57638731&dopt=GenPept&RID=U6BJJ99H015&log$=prottop&blast_rank=117) |
| *Scytonema sp.* | Bacteria, Cyanobacteria |  | AY768501 | [AAX44131](http://www.ncbi.nlm.nih.gov/entrez/query.fcgi?cmd=Retrieve&db=Protein&list_uids=61374283&dopt=GenPept&RID=U6BJJ99H015&log$=prottop&blast_rank=126) |
| *Scytonema sp.* | Bacteria, Cyanobacteria |  | AY768502 | [AAX44132](http://www.ncbi.nlm.nih.gov/entrez/query.fcgi?cmd=Retrieve&db=Protein&list_uids=61374285&dopt=GenPept&RID=U6BJJ99H015&log$=prottop&blast_rank=115) |
| *Stigmatella aurantiaca* | bacteria | StiC protein | AJ421825 | CAD19087 |
| *Stigmatella aurantiaca* | Bacteria, Proteobacteria |  | NZ_AAMD01000039 | [ZP_01462080](http://www.ncbi.nlm.nih.gov/entrez/query.fcgi?cmd=Retrieve&db=Protein&list_uids=115374805&dopt=GenPept&RID=U6BJJ99H015&log$=prottop&blast_rank=91) |
| *Stigmatella aurantiaca* | Bacteria, Proteobacteria |  | NZ_AAMD01000230 a | [ZP_01466715](http://www.ncbi.nlm.nih.gov/entrez/query.fcgi?cmd=Retrieve&db=Protein&list_uids=115379631&dopt=GenPept&RID=U6BJJ99H015&log$=prottop&blast_rank=79) |
| *Stigmatella aurantiaca* | Bacteria, Proteobacteria |  | NZ_AAMD01000230 b | [ZP_01466717](http://www.ncbi.nlm.nih.gov/entrez/query.fcgi?cmd=Retrieve&db=Protein&list_uids=115379633&dopt=GenPept&RID=U6BJJ99H015&log$=prottop&blast_rank=113) |
| *Stigmatella aurantiaca* | Bacteria, Proteobacteria |  | NZ_AAMD01000364 | [ZP_01467546](http://www.ncbi.nlm.nih.gov/entrez/query.fcgi?cmd=Retrieve&db=Protein&list_uids=115380596&dopt=GenPept&RID=U6BJJ99H015&log$=prottop&blast_rank=47) |
| *Streptomyces antibioticus* | Bacteria, Actinobacteria | ChlB1, Iterative type I | DQ116941 | AAZ77673 |
| *Streptomyces carcinostaticus* | Bacteria, Actinobacteria | ncsB, Iterative type I | AY117439 | AAM77986 |
| *Streptomyces pactum* | Bacteria, Actinobacteria | pctS, iterative type I, pactamycin | AB303063 | BAF92601 |
| *Streptomyces viridochromogenes* | Bacteria, Actinobacteria | aviM, Iterative type I, orsellinic acid | AF333038 | AAK83194 |
| *Streptomyces vitaminophilus* | Bacteria, Actinobacteria | Pyrrolomycin, pyr25 | EF140901 | [ABO15861](http://www.ncbi.nlm.nih.gov/entrez/query.fcgi?cmd=Retrieve&db=Protein&list_uids=126513523&dopt=GenPept&RID=U6BJJ99H015&log$=prottop&blast_rank=57) |
| *Strongylocentrotus purpuratus* | Eukaryota, Echinodermata | PKS | XM_788471 | gi115704918 |
| *Symploca sp.* | Bacteria , Cyanobacteria |  | AY604655 | AAU93832 |
| *Tetraodon nigroviridis* | Eukaryota, Vertebrata, Teleostei | PKS | CAAE01014768 | gi47221112 |
| *uncultured bacterium* | Bacteria | PKS7 | AJ639922 | CAG25974 |
| *uncultured bacterium* | Bacteria | Modular polyketide synthase | AY897138 | [AAW84168](http://www.ncbi.nlm.nih.gov/entrez/query.fcgi?cmd=Retrieve&db=Protein&list_uids=59149910&dopt=GenPept&RID=U6BJJ99H015&log$=prottop&blast_rank=102) |
| *uncultured bacterium* | Bacteria | Modular polyketide synthase | AY897142 | [AAW84172](http://www.ncbi.nlm.nih.gov/entrez/query.fcgi?cmd=Retrieve&db=Protein&list_uids=59149918&dopt=GenPept&RID=U6BJJ99H015&log$=prottop&blast_rank=88) |
| *uncultured bacterium* | Bacteria | Modular polyketide synthase | AY897146 | [AAW84176](http://www.ncbi.nlm.nih.gov/entrez/query.fcgi?cmd=Retrieve&db=Protein&list_uids=59149926&dopt=GenPept&RID=U6BJJ99H015&log$=prottop&blast_rank=127) |
| *uncultured bacterium* | Bacteria | Modular polyketide synthase | AY897167 | [AAW84197](http://www.ncbi.nlm.nih.gov/entrez/query.fcgi?cmd=Retrieve&db=Protein&list_uids=59149968&dopt=GenPept&RID=U6BJJ99H015&log$=prottop&blast_rank=107) |
| *uncultured bacterium* | Bacteria, Proteobacteria |  | DQ673146 | [ABG20990](http://www.ncbi.nlm.nih.gov/entrez/query.fcgi?cmd=Retrieve&db=Protein&list_uids=108794977&dopt=GenPept&RID=U6BJJ99H015&log$=prottop&blast_rank=105) |
| *Xylaria sp.* | Ascomycota, Sordariomycetes |  | DQ003485 | AAY46446 |
